# Medicolegal aspects of robotic-assisted surgery: litigation trends, complication risks, and human factors interventions

**DOI:** 10.1007/s11701-026-03685-y

**Published:** 2026-07-20

**Authors:** Robert A Keenan, Dara O Kavanagh, Sorcha O’Meara, Liam M Chadwick, Barry B McGuire, Fardod O’Kelly

**Affiliations:** 1 St Vincent’s University Hospital, Elm Park, Dublin 4, Ireland; 2 Department of Surgical Affairs, Royal College of Surgeons in Ireland, 121 St. Stephen’s Green, Dublin 2, Ireland; 3 Tallaght University Hospital, Dublin 24, Tallaght, Ireland; 4 Division of Paediatric Urology, Beacon Hospital Dublin, University College Dublin, Dublin, Ireland; 5 National Surgical Training Centre, Royal College of Surgeons in Ireland, 121 St. Stephen’s Green, Dublin, Ireland

**Keywords:** Human factors, Patient safety, Ergonomics, Robotics, Medicolegal

## Abstract

Robotic-assisted surgery (RAS) sees over six million procedures performed annually. Malpractice claims involving RAS increased by more than 250% between the periods 2006–2013 and 2014–2021 [1]. Understanding the legal landscape, risk profile, and evidence-based mitigation strategies is critical for all stakeholders. This scoping review aimed to: (1) map medicolegal cases involving RAS; (2) quantify complication incidence (3) characterise general and procedure-specific risks; and (4) synthesise interventions capable of reducing adverse events and litigation exposure. A structured search of PubMed/MEDLINE, EMBASE, the Westlaw legal database and FDA MAUDE was conducted for English-language sources from January 2000 to May 2026 [2]. Included sources reported medicolegal cases, adverse event rates, or human factors interventions in RAS. Sixty-one US malpractice cases were identified with 169 total liabilities claimed, most commonly negligent surgery, misdiagnosis, delayed treatment, and lack of informed consent [1]. Individual indemnity payments averaged $1,251,274. FDA MAUDE data (2000–2013) recorded 144 deaths, 1,391 injuries, and 8,061 device malfunctions across 1.75 million procedures [3]. Overall adverse event rates were 0.0834% [3]. Key modifiable risk factors include cognitive overload, loss of haptic feedback, team communication breakdown, and suboptimal ergonomics [4–7]. Medicolegal risk in RAS is rising but remains concentrated in a small number of procedure types and surgeon-experience brackets. The majority of claims relate to human error rather than device malfunction [1]. Severable actionable risk-reduction strategies such as structured training, governance, simulation-based competency are discussed [6, 8–10]. Adverse event registries and credentialing standards remain urgent legislative priorities.

## Introduction

Robotic-assisted surgery has undergone one of the most rapid technological adoptions in the history of procedural medicine. Since FDA clearance of the da Vinci Surgical System in 2000, the number of installed platforms has grown to over 7,000 globally, with more than six million procedures performed globally each year [1, 2]. The system’s three-dimensional, magnified visualisation, tremor filtration, and seven degrees of instrumental freedom have expanded minimally invasive options across urology, gynaecology, colorectal surgery, cardiothoracic surgery, head & neck surgery, and beyond [2, 3]. In real terms, this translates to improved ergonomics and reduced mechanical burden for the surgeon compared to traditional laparoscopic and open surgery, potentially encouraging career longevity [4, 5].

This technological enthusiasm has not, however, been without consequence. The FDA’s Manufacturer and User Facility Device Experience (MAUDE) database recorded 10,624 adverse event reports related to the da Vinci system between 2000 and 2013, encompassing 144 deaths, 1,391 patient injuries, and 8,061 device malfunctions (defined as software, mechanical or electrical failures before or during robotic procedures) across an estimated 1.75 million procedures [6]. Malpractice claims specifically involving RAS increased by more than 250% across 61 US court cases between 2006 and 2013 and 2014–2021 resulting in a mean indemnity payment of $1,251,274 [7].

The medicolegal landscape of RAS is complex. Unlike conventional open or laparoscopic surgery, liability in RAS may be shared among the operating surgeon, the institution, the bedside assistant, and the device manufacturer, a tripartite accountability structure with no settled legal precedent in many jurisdictions [8–10]. Simultaneously, the learning curve inherent to robotic platforms means that inexperienced surgeons may operate with suboptimal situational awareness, higher cognitive load, and reduced haptic feedback, all of which correlate with adverse outcomes [11, 12].

We hypothesize that malpractice risk in RAS is disproportionately concentrated among low-volume or early-learning-curve surgeons performing high-complexity procedures, and that this risk is driven primarily by modifiable human factors rather than device malfunction. This scoping review synthesises the available medicolegal literature, quantifies the incidence of specific adverse events, and evaluates the human factors and ergonomic interventions most likely to reduce both clinical harm and legal exposure.

## Methods

### Study design

This review was conducted in accordance with the Arksey and O’Malley scoping review framework (2005) and reported following the PRISMA-ScR extension for scoping reviews. The protocol was not pre-registered.

### Search strategy

Systematic searches were conducted across PubMed/MEDLINE, EMBASE, CINAHL, the Cochrane Library, and the Westlaw legal database. The FDA MAUDE database was searched directly. Search terms included: (“robotic surgery” OR “robot-assisted surgery” OR “da Vinci” OR “RARP” OR “robotic hysterectomy”) AND (“malpractice” OR “litigation” OR “medicolegal” OR “adverse event” OR “complication” OR “human factors” OR “ergonomics”). English-language sources published between January 2000 and May 2026 were eligible.

### Inclusion and exclusion criteria

Studies were included if they reported: medicolegal cases, verdicts, or settlements involving RAS; adverse event rates quantified per procedure or per console hour; or human factors/ergonomic interventions with measurable outcomes in RAS. Excluded were: non-English sources; studies exclusively concerning non-robotic minimally invasive surgery without comparative RAS data; case reports with fewer than three subjects; and opinion pieces without supporting data.

### Data extraction and synthesis

Data were extracted by two independent reviewers using a standardised form. Discrepancies were resolved by consensus. Given the heterogeneity of legal databases, clinical registries, and experimental human factors studies, a narrative synthesis was performed. Complication rates per 1,000 console hours were estimated by dividing event counts by estimated cumulative operating hours, using a conservative mean console time of 2.5 h per procedure for routine cases and 4.0 h for complex procedures [6, 11].

## Results

### Medicolegal landscape: case analysis

A total of 61 US malpractice cases were identified in the Westlaw database spanning 2006–2021 across 25 states, representing a 250% increase in the latter period compared to the former [7]. Defence verdicts predominated (77.8% in 2014–2021), but the financial consequences of plaintiff verdicts and settlements were substantial, with damages ranging from $10,087 to $5,008,922 (mean $1,251,274) [7]. Outside the US, European and Australian jurisdictions have seen product liability actions against Intuitive Surgical citing failure to warn and device defect, though lower litigation volumes reflect differences in healthcare funding structures, legal culture, and medical device regulation [8–10].

The most litigated specialties in the USA were obstetrics/gynaecology (48.9%), general surgery (28.9%), and urology (15.6%) [7]. The single most commonly litigated procedure was hysterectomy (42.2% of cases), followed by prostatectomy and hernia repair [7]. The most frequently claimed liabilities were negligent surgery (82.2%), misdiagnosis or failure to diagnose (46.7%) (perioperative management rather that direct related to procedure), delayed treatment (35.6%), and lack of informed consent (31.1%) with some litigations citing multiple liabilities: the latter a distinctively robotic-specific liability arising from failure to disclose the surgeon’s learning curve status [7, 12, 13]. Although no specific case details are provided, the theme of lack of informed consent appears to emanate from plaintiffs not fully understanding the surgical risks involved as well as the surgical experience and learning curve status of the robotic surgeon [13, 14] .

Selected illustrative cases are detailed in Table 1, with country of origin, procedure, patient outcome, claim type, quantum, and legal verdict.

**Table 1:** Selected medicolegal cases in robotic-assisted surgery (2006–2025)

Country	Robotic System	Procedure	Patient Outcome	Claim Type	Quantum (USD)	Legal Verdict
USA	da Vinci (IS)	Robotic hysterectomy	Death from sepsis (undetected bowel perforation)	Negligent surgery; failure to diagnose	$5,008,922	Plaintiff verdict
USA	da Vinci (IS)	Robotic myomectomy	Death — air embolism / cardiac arrest	Negligent surgery	$3,000,000	Plaintiff verdict
USA	da Vinci (IS)	Radical prostatectomy (RARP)	Chronic Regional Pain Syndrome; dysesthesia (Trendelenburg crush injury)	Negligent positioning	$950,000 settlement	Settlement
USA	da Vinci (IS)	Robotic hysterectomy (2022)	Death from sepsis (missed bowel leak)	Negligence (surgeon 40%; staffing agency 40%; hospital 20%)	$1,200,000	Plaintiff verdict
USA	da Vinci (IS)	Robot-assisted cholecystectomy	Liver laceration (spontaneous arm movement)	Device malfunction; improper use	$700,000 settlement	Settlement
USA	da Vinci (IS)	Robotic hysterectomy (most common procedure)	Variable (infection, retained FB, organ injury)	Negligent surgery (82%); lack of consent (31%)	$10,087–$5,008,922 (mean $1,251,274)	Mixed (77.8% defence verdicts)
USA	da Vinci (IS)	Robotic colectomy	Bowel injury; death	Device malfunction (electrical arcing)	Undisclosed	Settlement
USA	da Vinci (IS)	Radical prostatectomy	Urethral/bladder injury; incontinence	Negligent surgery; inadequate training	$400,000–$2,500,000 range	Mixed
Europe (multi)	da Vinci (IS)	Various (gynaecology, urology)	Organ injury; delayed diagnosis	Product liability; failure to warn	Undisclosed (EU regulatory framework)	Ongoing / settled
Australia	da Vinci (IS)	Robotic prostatectomy	Rectal injury; fistula	Negligent surgery; inadequate consent	Undisclosed	Settlement
USA	da Vinci (IS)	Robotic hysterectomy	Bowel perforation (2 sites; only 1 repaired)	Negligent surgery	$504,966	Plaintiff verdict (2025)
USA	da Vinci (IS)	Robotic nephrectomy	Vascular injury; haemorrhage	Negligent surgery; failure to convert	~$1,500,000	Settlement

IS = Intuitive Surgical; FB = foreign body; RARP = robot-assisted radical prostatectomy. Quantum figures in USD. EU cases reflect product liability claims under national medical device regulations. Cases sourced from Westlaw, Miller & Zois case database, and published medicolegal reviews [7, 10]

### Adverse event incidence: FDA MAUDE analysis

The most comprehensive adverse event data derive from Alemzadeh et al.‘s analysis of 14 years of MAUDE reports (2000–2013), encompassing an estimated 1.75 million robotic procedures [6]. During this period, 10,624 reports were filed, comprising 144 deaths (1.4%), 1,391 patient injuries (13.1%), and 8,061 device malfunctions (75.9%). The overall rate of injury or death events per procedure remained relatively stable from 2007 onwards at a mean of 83.4 per 100,000 procedures (95% CI: 74.2–92.7) [6].

Surgical specialties with the highest procedural volumes (gynaecology and urology) demonstrated lower adverse event rates (106.3 per 100,000) than less commonly robotically-adopted fields such as cardiothoracic and head/neck surgery (232.9 per 100,000; Risk Ratio = 2.2, 95% CI: 1.9–2.6) [6]. Device and instrument malfunction accounted for the majority of reports; the most common specific failure modes were fragments of burnt or broken instruments falling into the patient (14.7%), electrical arcing (10.5%), unintended instrument activation (8.6%), system/software errors (5.0%), and video or imaging problems (2.6%) [6, 15]. In 10.4% of all adverse events, the procedure was interrupted, requiring system restart (3.1%), conversion to non-robotic technique (7.3%), or rescheduling (2.5%) [7]. Gynaecology-specific MAUDE data from 2010 to 2020 identified instrument malfunction (predominantly accessories rather than the robot itself) as the principal cause, with most malfunctions having only minor impact on procedure completion [16].

Table 2 presents estimated complication incidence per 1,000 robotic console hours alongside rates per 100,000 procedures for the major adverse event categories.

**Table 2:** Estimated incidence of complications per 1,000 robotic console hours and per 100,000 procedures

Complication Type	Estimated Rate per 1,000 Console Hours*	Rate per 100,000 Procedures	Source / Notes
All adverse events (injuries + deaths)	~ 8.3	83.4 (95% CI 74.2–92.7)	Alemzadeh et al. 2016 (MAUDE 2000–2013)
Patient injury (any)	~ 1.1	13.1 per 10,000 reports	MAUDE database; 1,391/10,624 reports
Patient death	~ 0.12	1.4 per 10,000 reports	MAUDE; 144/10,624 reports
Device / instrument malfunction	~ 6.7	75.9% of all adverse event reports	MAUDE; includes arc, broken instruments, unintended activation
Electrical arcing of instruments	~ 0.87	10.5% of malfunction events	MAUDE 2000–2013
Broken/burnt instrument fragment in patient	~ 1.2	14.7% of malfunction events	MAUDE 2000–2013
Unintended instrument activation	~ 0.72	8.6% of malfunction events	MAUDE 2000–2013
System software/power failure	~ 0.42	5.0% of malfunction events	MAUDE 2000–2013
Video/imaging failure	~ 0.22	2.6% of malfunction events	MAUDE 2000–2013
Conversion to non-robotic / open technique	~ 0.61	7.3% of procedure interruptions	MAUDE; 7.3% of 1,104 interruptions
Vascular injury (major)	~ 0.08–0.17	1–2 per 100 procedures	Journal of Robotic Surgery; higher in learning-curve cases
Organ perforation (bowel, bladder, ureter)	~ 0.5–1.5	Varies by specialty: gynae 5–12%	Multiple case series; highest in colorectal (15–25%)
Cardiothoracic / head & neck complications	~ 2.0	232.9 per 100,000 procedures	Risk ratio 2.2 vs. gynae/urology (MAUDE)
Gynaecology / urology complications	~ 0.9	106.3 per 100,000 procedures	MAUDE; lower-complexity specialties
Anastomotic leak (colorectal)	~ 0.3–0.5	Approx. 2–5% (procedure-specific)	Meta-analyses of robotic vs. laparoscopic rectal surgery
Positioning injury (Trendelenburg)	~ 0.05–0.1	Case reports; nerve/crush injuries	Associated with prolonged steep Trendelenburg position

*Console hours estimated using mean 2.5 h per routine procedure and 4.0 h for complex procedures. Rates derived from FDA MAUDE database (2000–2013) [6], supplemented by specialty-specific meta-analyses [16–18]. Values represent best estimates; direct per-console-hour surveillance data are not available from any single primary source. FB = foreign body; IS = Intuitive Surgical

### General risks of robotic-assisted surgery

The overall complication rate for RAS is procedure- and specialty-dependent but compares favourably with open surgery across most domains. For radical prostatectomy, RARP carries an overall perioperative complication rate of 7.8% versus 11.1% for laparoscopic and 17.9% for open approaches [17]. For hysterectomy, reoperation rates within 30 days are lower for robotic (2.5%) versus abdominal approaches (4.1%) [17]. For colorectal surgery, meta-analyses of randomised controlled trials show reduced surgical site infection rates reduced blood loss and reduced all-cause mortality between robotic and laparoscopic rectal surgery, with robotic surgery demonstrating significantly lower conversion rates to open technique [19].

Despite these overall favourable comparisons, several generic risks are amplified in the robotic context:

- Loss of haptic feedback: with the exception of the latest Da Vinci model, DV5, released in 2024, no robotic platforms provide tactile force feedback, requiring surgeons to rely entirely on visual cues to gauge tissue tension. This increases the risk of inadvertent vessel or organ injury, particularly during the learning curve [11, 13].
- Steep Trendelenburg positioning: Prolonged pneumoperitoneum in steep Trendelenburg, essential for pelvic procedures, is associated with increased intraocular pressure, brachial plexus injury, compartment syndrome, and facial/airway oedema [20, 21]. The overall incidence of positioning-related transient neurological insult is estimated at 2–5% [21].
- Delayed recognition of complications: The physical separation of the surgeon from the operative field at the console may reduce situational awareness, delaying recognition of intraoperative haemorrhage or perforation [22, 23].
- Instrument insulation failure: Robotic instruments with compromised insulation may conduct electrical current to inadvertent tissue sites, causing thermal injury at sites remote from the operative field [24].
- Learning curve vulnerability: The majority of serious complications occur during the learning curve phase. Procedure specific risks are discussed in detail below. Estimates suggest 150–250 cases are required to achieve proficiency in RARP [25, 26] This common but complex procedure is primarily for oncological purposes however crucial components include neurovascular bundle dissection, essential for erectile function preservation, as well as urethral dissection and reconstruction which is important for regaining urinary continence postop. Therefore some authorities cite over 800 cases are required for RARP mastery although this would be considered at the more complex end of the spectrum of robotic procedures in comparison to robotic hysterectomy which requires approximately 91 procedures for mastery [27, 28].
- Team dynamics and communication failure: The console surgeon’s physical separation from the bedside team impairs real-time communication, instrument exchanges, and coordinated emergency response [22, 23, 29].

### Procedure-specific risks

#### Urological procedures

RARP is the most frequently performed robotic procedure globally and the most litigated urological operation. Vascular injury, including injury to the dorsal venous complex, obturator vessels, and iliac vessels during trocar placement, occurs in approximately 1–2% of cases and is disproportionately concentrated in the first 50 cases of a surgeon’s series [30]. Urethral anastomotic insufficiency and rectourethral fistula, though rare (< 1%), carry devastating functional consequences and significant medicolegal risk. Robot-assisted radical cystectomy (RARC) carries a longer operative time than open surgery with similar complication profiles although lower wound complications, venous thromboembolic events, blood loss and less readmissions [31].

#### Gynaecological procedures

Robotic sacrocolpopexy carries risks of vascular injury to middle sacral vessels (< 1%) and bowel injury (0–8%) [32]. Hysterectomy is the single most litigated robotic procedure [7]. Bladder and ureteric injuries occur in 1–3% of cases. Vaginal cuff dehiscence, a complication unique to minimally invasive hysterectomy, has an incidence of approximately 1.6% in robotic series, compared to 0.64–1.35% in laparoscopic series, and is associated with colpotomy technique and energy use [33]. Critically, the LACC trial (2018) demonstrated inferior oncological outcomes for minimally invasive radical hysterectomy in cervical cancer, resulting in a practice-changing shift and subsequent medicolegal scrutiny of cases performed prior to the trial’s publication [34].

#### Colorectal and general surgery

Robotic colorectal surgery carries anastomotic leak rates of 2–5%, comparable to laparoscopic and open approaches but associated with more severe consequences owing to the prolonged operative time and delayed recognition of haemorrhage in the console-isolated surgeon [19]. Robotic cholecystectomy has been the subject of litigation following cases of bile duct injury and intraoperative arm malfunction causing hepatic laceration [8]. Robotic hernia repair has seen an over eight-fold increase in case volume since 2010, compared to a three-fold increase and a 0.6-fold decrease in the laparoscopic and open approaches respectively despite showing the highest long-term incidence of operative recurrences of 3.78% [35].

#### Cardiothoracic and head & neck surgery

These specialties carry the highest adverse event rates per procedure in the MAUDE database (232.9 per 100,000 versus 106.3 for gynaecology/urology; RR = 2.2) [6]. This is attributed to the procedural complexity, less frequent use of robotic platforms reducing cumulative operator experience, and the limited tolerance for intraoperative error in cardiac surgery [6, 36]. Robotic mitral valve repair and thoracoscopic lobectomy represent the highest-risk operative contexts within the RAS landscape [6].

### Human factors and ergonomic interventions

#### Overview of human factors in robotic surgery

Human Factors science examines the interaction between humans, technology, and work systems. In RAS, the operating surgeon at the console, the bedside assistant, the scrub nurse, and the anaesthetic team each occupy a distinct technological and spatial role, creating multiple points of potential system failure [29]. Although RAS reduces physical ergonomic strain for the console surgeon compared to open or laparoscopic surgery, it has also been found to reduce cognitive strain (NASA-TLX score RAS 32.4 ± 10.3 SD vs. laparoscopic 45.6 ± 14.3 SD [*p* < 0.001] [37]. Yet it simultaneously introduces new cognitive and communicative challenges that have been insufficiently addressed in training programmes [11, 20].

#### Cognitive workload

Cognitive workload (CWL) in RAS is multidimensional, comprising intrinsic load (dependent on procedure complexity and surgeon expertise), extraneous load (interface design and interruptions), and germane load (learning) [11]. Experienced robotic surgeons operating in their domain of expertise demonstrate automaticity, the capacity to perform technical tasks without occupying full working memory, enabling superior error detection and correction under elevated task complexity [11, 38].

Conversely, surgeons transitioning from laparoscopic to robotic platforms without structured training experience elevated CWL from loss of established motor schemas, unfamiliar instrument kinematics, reduced haptic feedback, and the cognitive demands of visual compensation for missing tactile information [11]. EEG studies have demonstrated alpha and beta desynchronisation, markers of mental fatigue, during robotic cases in surgeons without prior robotic experience [11]. Workflow disruptions (instrument exchanges, system reboots, communication interruptions) further increase CWL and have been independently associated with surgical error in robotic colorectal and gynaecological surgery [38].

Interventions to reduce CWL include:

- Structured simulation training with progressive task difficulty, enabling automaticity before exposure to clinical cases [4, 25].
- Standardised operative checklists and ‘time-out’ protocols specific to RAS, addressing docking configuration, instrument checks, and emergency conversion pathways [39].
- Real-time CWL monitoring using physiological metrics (heart rate variability, pupillometry, EEG) to identify at-risk operative moments and prompt team support [11].
- Reduction of non-essential intraoperative communication and avoidance of elective workflow interruptions during high-complexity operative phases [38].
- A system of proctoring, either internal, external, or peer-to-peer mentoring has been shown to be highly sought-after for robotic surgeons as a means of sharing the cognitive load of a case, particularly when a surgeon is in the vulnerable early stages of their learning curve and mitigates the impact of this learning curve on patient outcomes [40, 41].
- FUSE (Fundamental Use of Surgical Energy) training and simulation to improve the knowledge and use of electrosurgery with the aim to reduce arcing and electrosurgical errors [42].

#### Physical ergonomics

The da Vinci console provides a seated, wrist-supported operating position with scaled motion and tremor filtration, substantially reducing the musculoskeletal (MSK) strain associated with laparoscopic surgery [20, 21]. However, suboptimal console ergonomics, including incorrect seat height, footrest position, and eyepiece alignment, remain prevalent even among experienced surgeons and are associated with neck strain, lower back pain, and eye fatigue [21].

Bedside assistants in RAS experience greater musculoskeletal strain than their laparoscopic counterparts, owing to awkward instrument positioning necessitated by the robot’s fixed arm configuration, with up to 73% reporting uncomfortable working conditions and 20% reporting pain or bruising [43]. Standardised ergonomic assessment of the bedside position, including monitor height, instrument table placement, and foot position, is recommended as a component of every RAS team briefing [29].

Evidence-based physical ergonomic interventions include:

- Pre-case ergonomic assessment and console configuration tailored to individual surgeon anthropometry [21].
- Use of validated ergonomic scoring tools (e.g., RULA — Rapid Upper Limb Assessment) for bedside assistant posture evaluation [21].
- Mandatory rest breaks for procedures exceeding three console hours to prevent fatigue-related performance degradation [11, 21].
- Ergonomic hand positioning training to correct visual-motor mismatch during robotic colorectal procedures [11].
- Consider using a robotic platform that uses an open console design, such as the Hugo RAS (Medtronic), Versius (CMR), KangDuo (Harbin Sagebot) or Mantra (SSi), where there is some emerging evidence that an open console platform can alleviate MSK strain while achieving equivalent technical performance [44].

#### Team communication and situational awareness

The physical separation of the console surgeon from the operative field, typically 2–5 m, introduces a significant barrier to the real-time situational awareness that characterises safe open or laparoscopic surgery [22, 23]. The console surgeon’s restricted field of view, combined with attentional focus at the operative site, may cause delayed recognition of patient deterioration signals communicated from the anaesthetic team although this may be mitigated in cases utilising an open-console such as Medtronic’s Hugo RAS [45].

Communication failures in the robotic operating room have been categorised as: (a) surgeon-to-bedside assistant communication during instrument exchanges and emergencies; (b) surgeon-to-anaesthetist communication about haemodynamic tolerance of positioning and pneumoperitoneum; and (c) team-to-team communication during system malfunction and emergency conversion [23, 29, 30].

High-reliability interventions from aviation and nuclear industries that are applicable to RAS include:

- Crew Resource Management (CRM) training adapted for robotic theatre teams, emphasising assertive communication, closed-loop confirmation, and shared mental models [46].
- Structured RAS-specific team briefings before every case, covering emergency conversion plan, instrument malfunction response, and patient-specific risk factors [39].
- Voice-activated communication systems allowing console surgeons to issue commands and receive updates without losing operative concentration [22].
- Mandatory emergency conversion drills conducted at regular intervals to maintain team readiness [23, 39].

#### Training, credentialing, and the learning curve

The absence of universal, mandatory credentialing standards for RAS represents the most significant and most readily actionable contributor to medicolegal risk [4, 12, 39]. In the United States, Intuitive Surgical has historically provided manufacturer-led training without legal authority to mandate certification before commercial sale of the system [10]. Plaintiff attorneys have successfully argued that this creates liability for institutions that permit surgeons to perform robotic procedures without verified competency [12, 13].

The Society of Urologic Robotic Surgeons recommends a minimum of three to five proctored cases before independent practice, with ongoing volume requirements of 20–24 procedures per year to maintain credentialing [39]. The European Society of Coloproctology’s 2024 guideline specifies structured multi-stage training including robotic simulation, dry laboratory, wet laboratory, proctored clinical cases, and formal competency assessment before unsupervised practice [47]. The OCERT consensus confirmed that prior laparoscopic experience, simulation training, and structured proctorship substantially shorten the learning curve — from over 150 cases to as few as 12–15 cases for selected procedures [25].

Critically, informed consent for RAS must include explicit disclosure of the operating surgeon’s procedure-specific experience and current position on the learning curve, a requirement that remains inconsistently implemented and a source of growing litigation [13].

## Discussion

This scoping review demonstrates that medicolegal risk in RAS is rising, procedure-concentrated, and predominantly attributable to human error rather than device malfunction. The MAUDE database reveals that device and instrument malfunction accounts for 75.9% of adverse event reports, yet the majority of legal claims are filed for negligent surgery, misdiagnosis, and failure to obtain adequate informed consent, none of which are primarily technology failures [6, 7]. This distinction is crucial for risk management. It indicates that the primary levers for reducing both patient harm and legal exposure lie in the domains of surgical training, governance, team communication, and consent quality, not exclusively in device improvement. That said, persistent device malfunctions modes such as electrical arcing (10.5% of malfunction events) and unintended instrument activation (8.6%) represent tractable engineering problems that manufacturers have a responsibility to address and a legal duty to warn about [6, 15, 24]. Mitigation factors such as FUSE education as discussed in 3.5.2, aim to address the awareness of robotic surgeons to these potential sources of patient injury and take actions to reduce them.

The complication rates presented in Table 2 must be interpreted with caution. The MAUDE database is subject to significant underreporting bias, it captures only complications that are reported by manufacturers, facilities, and patients, with no denominator of total procedures by specialty until 2013 [6, 48]. The per-console-hour estimates derived in this review represent a novel metric not available from any single primary source, and should be validated prospectively in future national registries.

Human Factors research has produced actionable, evidence-graded interventions for cognitive workload reduction, ergonomic optimisation, and team communication improvement [11, 23, 29, 30]. However, implementation remains fragmented. Few institutions have adopted systematic ergonomic assessments, and CRM training for robotic theatre teams remains a rarity outside high-volume academic centres [49]. Furthermore, many institutions lack a defined governance strategy for the safe introduction and running of a robotic surgical programme within their hospitals. The Royal College of Surgeons in Ireland (RCSI) have formed the Robotic Surgical Leads Group in response to this, consisting of representatives from all surgical specialities using robotic platforms as well as quality assurance representatives to develop a guide for best practice for robotic surgery. This outlines recommendations for training, accreditation and governance for robotic surgery, with specific guides for patient safety around the surgeon learning curve, emergency scenarios and preparedness, and key performance indicator recommendations to ensure a safe, equitable and successful robotic programme [50].

International variation in the medicolegal framework for RAS creates asymmetric risk for global manufacturers and surgeons. European product liability law under the EU Medical Devices Regulation (MDR 2017/745) imposes stricter post-market surveillance and adverse event reporting requirements than the 510(k) pathway under which the da Vinci system received FDA clearance [9, 10]. This regulatory divergence will increasingly affect the global litigation landscape as robotic surgery volumes rise in Asia, Australia, and low- and middle-income countries [1, 51].

## Conclusions

Robotic-assisted surgery offers measurable clinical advantages in selected procedures, but carries a distinct and rising medicolegal risk profile shaped by inadequate training standards heretofore defined by industry, imperfect human-machine interaction, and inconsistent informed consent practices. Human factors interventions including structured simulation-based training, cognitive load optimisation, physical ergonomic assessment, and Crew Resource Management for robotic theatre teams, represent the most evidence-graded and actionable risk reduction strategies currently available. Universal mandatory governance of robotic teams and surgeons, procedure-specific informed consent disclosing surgeon learning curve status, and prospective national adverse event registries are urgently needed legislative and regulatory priorities.

## Data Availability

No datasets were generated or analysed during the current study.
